# The Role of Imported Cases and Favorable Meteorological Conditions in the Onset of Dengue Epidemics

**DOI:** 10.1371/journal.pntd.0000775

**Published:** 2010-08-03

**Authors:** Chuin-Shee Shang, Chi-Tai Fang, Chung-Ming Liu, Tzai-Hung Wen, Kun-Hsien Tsai, Chwan-Chuen King

**Affiliations:** 1 Graduate Institute of Epidemiology, College of Public Health, National Taiwan University, Taipei, Taiwan; 2 Department of Internal Medicine, National Taiwan University Hospital, Taipei, Taiwan; 3 Global Change Research Center, National Taiwan University, Taipei, Taiwan; 4 Department of Atmospheric Sciences, National Taiwan University, Taipei, Taiwan; 5 Department of Geography, National Taiwan University, Taipei, Taiwan; Colorado State University, United States of America

## Abstract

**Background:**

Travelers who acquire dengue infection are often routes for virus transmission to other regions. Nevertheless, the interplay between infected travelers, climate, vectors, and indigenous dengue incidence remains unclear. The role of foreign-origin cases on local dengue epidemics thus has been largely neglected by research. This study investigated the effect of both imported dengue and local meteorological factors on the occurrence of indigenous dengue in Taiwan.

**Methods and Principal Findings:**

Using logistic and Poisson regression models, we analyzed bi-weekly, laboratory-confirmed dengue cases at their onset dates of illness from 1998 to 2007 to identify correlations between indigenous dengue and imported dengue cases (in the context of local meteorological factors) across different time lags. Our results revealed that the occurrence of indigenous dengue was significantly correlated with temporally-lagged cases of imported dengue (2–14 weeks), higher temperatures (6–14 weeks), and lower relative humidity (6–20 weeks). In addition, imported and indigenous dengue cases had a significant quantitative relationship in the onset of local epidemics. However, this relationship became less significant once indigenous epidemics progressed past the initial stage.

**Conclusions:**

These findings imply that imported dengue cases are able to initiate indigenous epidemics when appropriate weather conditions are present. Early detection and case management of imported cases through rapid diagnosis may avert large-scale epidemics of dengue/dengue hemorrhagic fever. The deployment of an early-warning surveillance system, with the capacity to integrate meteorological data, will be an invaluable tool for successful prevention and control of dengue, particularly in non-endemic countries.

## Author Summary

Dengue/dengue hemorrhagic fever is the world's most widely spread mosquito-borne arboviral disease and threatens more than two-thirds of the world's population. Cases are mainly distributed in tropical and subtropical areas in accordance with vector habitats for *Aedes aegypti* and *Ae. albopictus*. However, the role of imported cases and favorable meteorological conditions has not yet been quantitatively assessed. This study verified the correlation between the occurrence of indigenous dengue and imported cases in the context of weather variables (temperature, rainfall, relative humidity, etc.) for different time lags in southern Taiwan. Our findings imply that imported cases have a role in igniting indigenous outbreaks, in non-endemics areas, when favorable weather conditions are present. This relationship becomes insignificant in the late phase of local dengue epidemics. Therefore, early detection and case management of imported cases through timely surveillance and rapid laboratory-diagnosis may avert large scale epidemics of dengue/dengue hemorrhagic fever. An early-warning surveillance system integrating meteorological data will be an invaluable tool for successful prevention and control of dengue, particularly in non-endemic countries.

## Introduction

Dengue outbreaks initiated by international tourists, immigrants, and foreign workers have been reported in numerous developed areas and countries [1], [2], [3]. Nevertheless, the interplay between infected travelers, climate, vectors, and indigenous dengue incidence remains unclear [3], [4].

**Figure 1:**
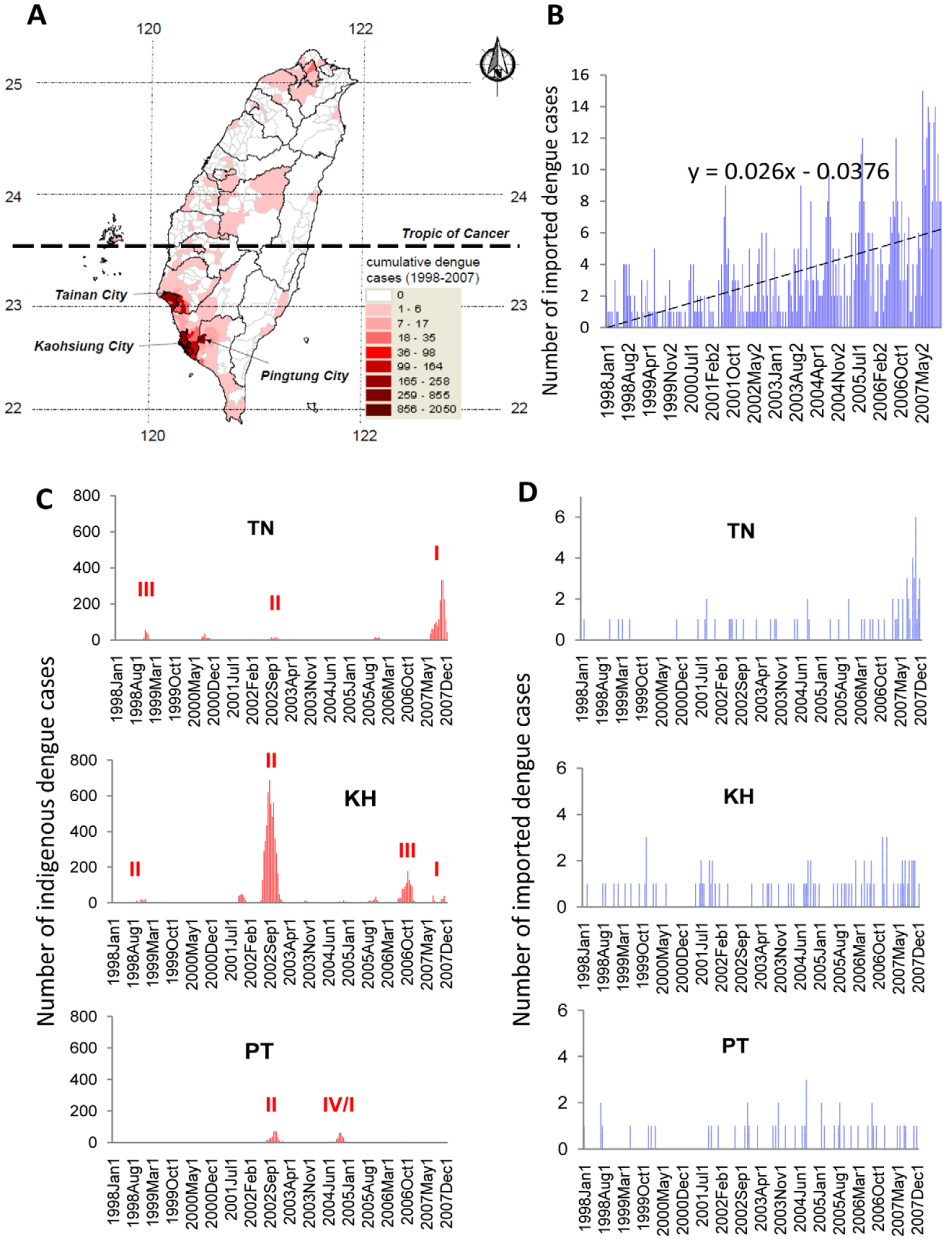
Numbers of laboratory-confirmed dengue (including dengue and dengue hemorrhagic fever) cases in Taiwan, 1998–2007. **A. Spatial distributions of cumulative indigenous dengue cases.** **B. Biweekly number of imported dengue cases.** **C. Biweekly number of indigenous dengue cases in the studied areas.** TN: Tainan area, including Tainan City and County; KH: Kaohsiung area, including Kaohsiung City and County; PT: Pingtung area, including Pingtung City and County. The Roman numbers denote predominant serotypes of dengue virus isolated during major epidemics in that area [10]. **D. Biweekly number of imported dengue cases in the studied areas.**

Historically, the link between imported cases and indigenous cases has been established through phylogenetical analysis and viral sequence comparisons [5], [6]. However, these retrospective studies are not capable of providing timely, relevant information about transmission dynamics, nor do they provide quantitative insight for disease control strategies in a broader context. For example, epidemiological data has indicated that imported dengue cases enter Taiwan almost every month from other countries (Figure 1B) but have not always resulted in local dengue epidemics [6], [7]. This suggests that the timing of imported dengue's entrance may have considerable effect on domestic dengue epidemics [3], [8]. However, the role of these foreign-origin cases in local dengue epidemics has not yet been quantitatively assessed [9].The aims of this study were to clarify the relationship between imported dengue, local weather, and domestic epidemics of dengue, and to further identify the role of imported cases (in different phases) during a dengue epidemic in non-endemic areas such as Taiwan.

The study used data of all imported and indigenous dengue cases nationwide that had been confirmed by the Centers for Disease Control in Taiwan (Taiwan-CDC) [10], [11], Republic of China (R.O.C.) to investigate the relationship between imported and indigenous dengue, and all concurrent meteorological characteristics with potential for facilitating disease transmission.

## Methods

### Surveillance of dengue

Dengue, including dengue fever (DF) and dengue hemorrhagic fever (DHF), are notifiable infectious diseases to be reported within 24 hours in Taiwan. Information on these confirmed cases of dengue fever (DF) and dengue hemorrhagic fever (DHF) were obtained from Taiwan-CDC from 1998 to 2007 through dengue surveillance in Taiwan. This surveillance system is made up of three parts: passive, active and semi-active surveillance. In passive surveillance, dengue-like illness reports by health care workers to local health authorities account for most confirmed dengue cases. Active surveillance, including volunteer reporting and fever screenings at international airports (identifying fever cases by infrared thermal scanner, which has been routinely operated by the government since 2003) [5], [6]. In semi-active surveillance, fever cases are investigated in residential areas, schools, and work places with epidemiological linkage, and specimens are taken once confirmed dengue cases are identified. These active and semi-active components, serve to complement and reinforce in support of comprehensive virus detection. Among active strategies, fever screening detects imported dengue cases efficiently [6]. All febrile patients identified through fever screening are required to submit blood samples for testing. In addition, public health professionals at local health authorities monitor suspected cases for the development of dengue-like symptoms/signs until dengue virus infection is excluded [10]. These strategies identify and manage potential dengue cases before they enter into the community.

### Case definition of dengue

The current definitions for dengue, including DF, DHF and dengue shock syndrome (DSS) in Taiwan have been applied since the 1980s. Cases of “probable DF” are patients with body temperatures ≥38°C and two or more of the following clinical manifestations: headache, retro-orbital pain, myalgia, arthralgia, rash, hemorrhagic manifestations and leucopenia. Cases of “probable DHF” and DSS are further identified based on criteria established by the World Health Organization [12]. Identified probable dengue cases must provide blood specimens for laboratory confirmation tests. These laboratory tests include molecular identification of dengue virus by reverse-transcriptase polymerase chain reaction (RT-PCR) [13], single or paired serum samples testing for dengue-specific IgM seropositives, 4-fold dengue-specific IgG serotiter rises (with the exclusion of Japanese encephalitis virus infection) [12], or virus isolation [14], [15]. Date of onset of dengue illness, age, gender, clinical manifestations, reporting hospital, and laboratory results were all thoroughly documented for each dengue case.

### Definition of imported dengue cases

Epidemiological questions such as travel history, incubation period, and first day of illness were evaluated to identify the possible origin of dengue infection. “Imported dengue cases into Taiwan” were defined as laboratory-confirmed dengue cases with travel history to endemic countries within 14 days before the date of onset of dengue (based on Taiwan-CDC's definition) [10].

### Study areas

Confirmed indigenous dengue cases in three epidemic areas in Southern Taiwan [Tainan (TN), Kaohsiung (KH), and Pingtung (PT)] were investigated. All three areas had identified both *Aedes aegypti* and *Ae. albopictus* mosquitoes as vectors for transmitting dengue virus. KH, including both metropolitan Kaohsiung and Kaohsiung County, had served as the location for the majority of Taiwan's dengue epidemics involving all four serotypes of dengue viruses. Smaller scale epidemics of dengue also occurred in both TN and PT, located adjacent to Kaohsiung. For this study, TN included Tainan City and County, while PT referred to Pingtung City and County. The subtropical climate of southern Taiwan presents an annual hot and rainy summer season lasting from June to August and daily mean temperatures ranging from 18° to 32°C year round.

### Serotype of dengue viruses in major epidemics

Information on the predominant serotype of isolated dengue viruses in TN, KH and PT (Figure 1C) was obtained from Taiwan-CDC [10]. Taiwan's dominant serotypes/genotypes of epidemic DENV varied by year and area [6]. However, in 2002, a DENV-2 epidemic attacked all three areas of our study. During our study period, KH had the most frequent occurrence of dengue epidemics, with epidemics of DENV-2 in 1998 and 2001–2003; DENV-1 in 2004; DENV-3 in 2006, and DENV-1 in 2007. TN had four major epidemics, including DENV-3 in 1998 [16], DENV-4 in 2000, DENV-2 in 2002, and DENV-1 in 2007. PT had two major epidemics, including DENV-2 in 2001–2003 and DENV-1/DENV-4 in 2004. We found that local dengue epidemics, with geographical variations in these three areas, only had higher numbers of indigenous cases during certain years.

### Meteorological data

We systematically collected daily weather data for Taiwan that was publicly available through the 26 branch stations of the Central Weather Bureau (http://www.cwb.gov.tw/). The meteorological variables analyzed in this study were selected after comprehensive evaluation of all available data with biological relevance to vectors or cases of dengue, including daily mean temperature, daily maximum temperature, daily minimum temperature, daily mean relative humidity, daily mean wind speed, daily accumulative rainfall, daily accumulative rainy hours, daily sunshine accumulation hours, daily mean sunshine rate (from sunrise to sunset), and daily sunshine total flux. Unlike weather stations in Tainan and Kaohsiung, Pingtung County's station is located a far distance from Pingtung City, where the majority of Pingtung's dengue cases occurred. We therefore used weather data collected by the Environment Protecting Agency (EPA) at their station in Pingtung City. This EPA weather station was only able to provide data regarding daily mean temperature, daily maximum temperature, daily minimum temperature, daily mean wind speed, and daily accumulative rainfall. We then substituted Kaohsiung's data for Pingtung's meteorological variables not provided by the EPA because of Pingtung City's close proximity to Kaohsiung City.

### Statistical analyses

All laboratory-confirmed daily dengue cases, according to the date of onset of dengue illness, were summed into total case numbers in bi-weekly intervals for data analysis. The mean value of each meteorological variable was also calculated for each biweekly interval. Abbreviations of all variables analyzed are listed in the Table 1. As the effects of imported dengue and meteorological factors on indigenous dengue logically had a time lag, we thus tested different time lags for each variable from lag 1 up to lag 12 (lag 1 representing two weeks, lag 2 representing four weeks, and so on).

**Table 1:** Abbreviations of variables.

Abbreviation	Explanation	Unit
**Independent variables**		
imported	number of imported dengue cases in the area	cases
rain	daily accumulative rainfall	mm
rainhr	daily accumulative rainy hours	hours
rh	daily mean relative humidity	%
sunhr	daily sunshine accumulation hour	hours
sunrate	daily mean sunshine rate (from sunrise to sunset)	%
tmax	daily maximum temperature	°C
tmean	daily mean temperature	°C
tmin	daily minimum temperature	°C
area_TN, area_KH	dummy variables: for cases from Tainan (TN) area, area_TN = 1 and area_KH = 0; for cases from Kaohsiung (KH) area, area_TN = 0 and area_KH = 1; cases from Pingtung (PT) area, area_TN = 0 and area_KH = 0)	none
popd	area-specific population density	population/km^2^
sin24	Oscillation function sin (2πt/T), T (period) = 12 months	none
cos24	Oscillation function cos (2πt/T), T (period) = 12 months	none
**Dependent variables**		
Occurrence (Figure 2)	Occurrence = 1 when any new indigenous confirmed dengue cases were present in the studied bi-week intervals, else Occurrence = 0.	none
Increase (Figure 3)	Increase = 1 when relative risk = [(number of indigenous dengue cases in the studied bi-week interval+0.5)/(number of indigenous cases in the prior interval+0.5)] was larger than 1.2^#^, else Increase = 0.	none
Case (Figure 4)	the number of indigenous dengue per bi-week in an area	cases/bi-week

^#^: The threshold of 1.2 was chosen for optimizing the apportionment ratio, in order to increase statistical efficiency. Use of alternative threshold, such as 1.5 or 2.0, decreased statistical efficiency for the analysis. Because that the number of indigenous cases per area during a bi-week in low transmission season is mostly zero, we calculate the ratio by adding 0.5 to both the denominator and numerator.

Logistic regression was used to analyze the correlation between the occurrence/increase of indigenous dengue and the number of imported cases, as well as the correlation between the occurrence/increase of indigenous dengue and each meteorological variable across various time lags (from 2 weeks to 24 weeks). Poisson regression was used to analyze the correlation between the number of indigenous dengue cases and the number of imported cases, as well as the correlation between the number of indigenous dengue cases and quantitative data of each meteorological variable across time lags from 2 weeks to 24 weeks. Regression with the negative binomial model [17] was used for over-dispersed data. All models were adjusted by area (two dummy variables, area_KH and area_TN), popd (area-specific population density), and sine24 plus cosine24 (the oscillatory *sine* and *cosine* functions were used to model seasonal variations of dengue cases [18] ).

Because the quantitative relationship between indigenous and imported dengue cases may exist only at the onset of local dengue epidemics, we further divided all bi-week intervals into three categories for further analysis: 1) **Period of “low intensity transmission”**: From March to May during our study period, 94.44% (170/180) of bi-week intervals during these three months had no indigenous dengue cases in these studied areas. 2) **Period of “early phase of outbreaks”**: Those bi-week intervals presenting <10 indigenous dengue cases for months excluding March to May. 3) **Period of “late phase of outbreaks”**: Those bi-week intervals presenting ≧10 indigenous dengue cases.

Further information on these regression models are listed in the Text S1. Two-tailed p<0.05 was regarded as statistically significant. The statistical analysis was conducted using S-PLUS Enterprise Developer Version 8.0.4 (TIBCO Software Inc., Palo Alto, CA, USA) and SAS 9.1.3 Service Pack 4 (SAS Institute Inc., Cary, NC, USA).

## Results

### Patterns of confirmed imported and indigenous dengue cases from 1998 to 2007

Among the 9,910 laboratory-confirmed indigenous dengue cases (mean age ± standard deviation = 45.63±18.67 years) from 1998 to 2007, 9,195 (92.79%) were adults (>15 years old). Cases in study areas accounted for 98.45% (9,756/9,910) of total confirmed indigenous cases in Taiwan during this period (Figure 1A). Figure 1B indicates that the number of biweekly imported dengue cases in Taiwan significantly increased over time (β = 0.026±0.002, p<0.0001). Comparing local dengue case numbers of the three study areas (TN, KH and PT) in Figure 1D with all of Taiwan's dengue cases in Figure 1B, Figure 1D serves to illustrate that imported dengue cases entered southern Taiwan almost every month (within one year) without a clear pattern. Indigenous dengue cases, in contrast, exhibited a strong seasonal regularity (Figure 1C).

### Logistic regression models for the occurrence and increase of indigenous dengue cases

**Figure 2:**
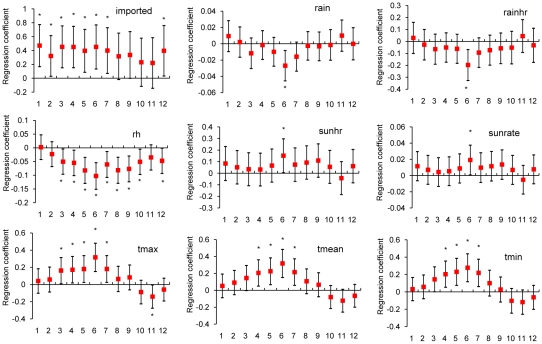
Correlation between bi-weekly “occurrence” of indigenous dengue cases and 1) the number of imported cases as well as 2)meteorological variables across time lags from 1 to 12 bi-weeks. Note: 1. Each vertical line segment corresponds to a 95% confidence interval of the regression coefficient and the red solid squares indicate the value of each coefficient estimate. (*: p<0.05). 2. The X-axis displays different time lags: 1 for two weeks lag, 2 for four weeks lag, and so on. 3. Abbreviations: see Table 1.

**Figure 3:**
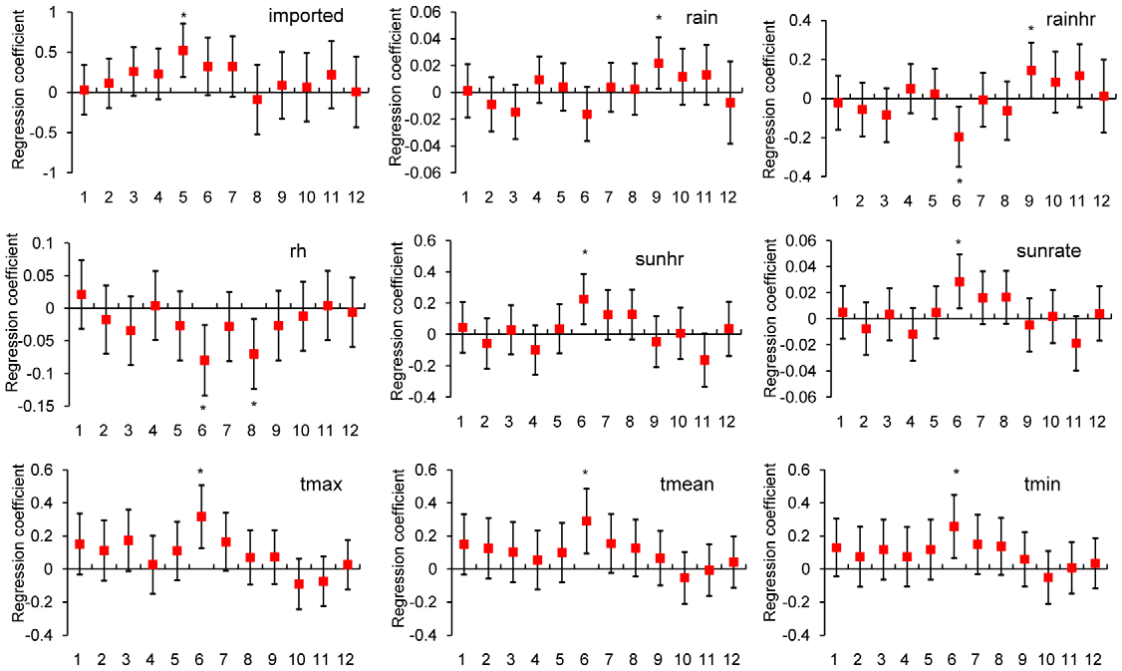
Correlation between bi-weekly “increase” of indigenous dengue cases and 1) the number of imported cases as well as 2)meteorological variables across time lags from 1 to 12 bi-weeks. Note: 1. Each vertical line segment corresponds to a 95% confidence interval of the regression coefficient and the red solid squares indicate the value of each coefficient estimate. (*: p<0.05). 2. The X-axis displays different time lags: 1 for two weeks lag, 2 for four weeks lag, and so on. 3. Abbreviations: see Table 1.

**Figure 4:**
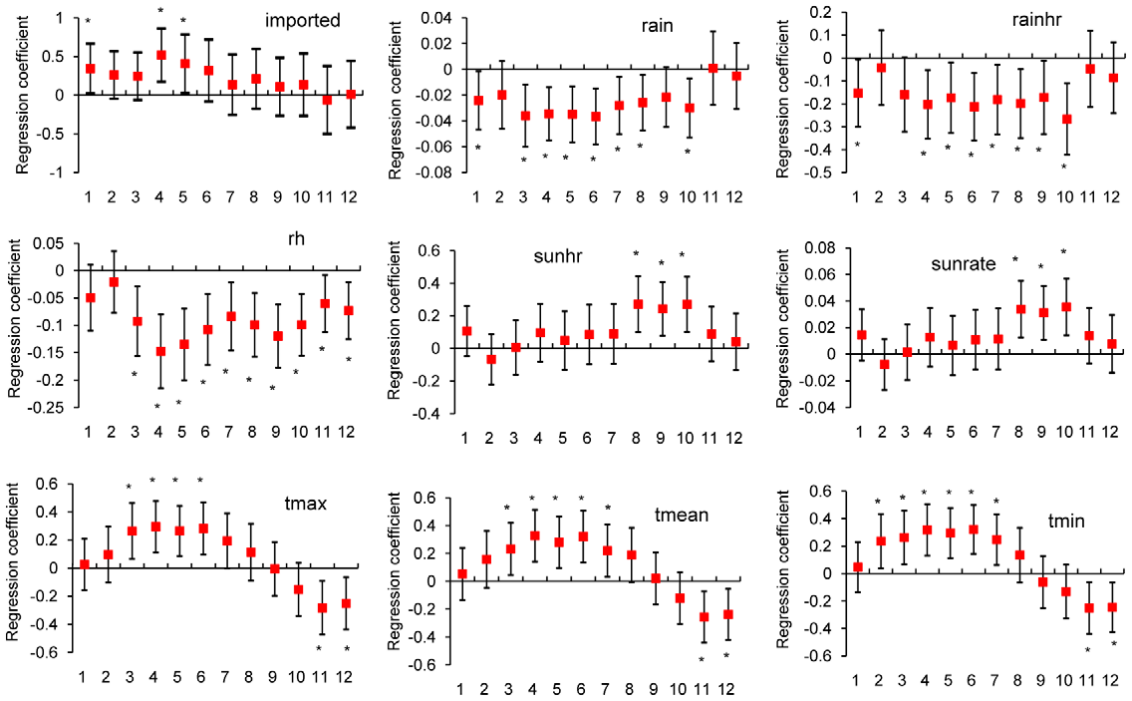
Correlation between bi-weekly number of indigenous dengue cases and 1) the number of imported cases as well as 2)meteorological variables across time lags from 1 to 12 bi-weeks. Note: 1. Each vertical line segment corresponds to a 95% confidence interval of the regression coefficient and the red solid squares indicate the value of each coefficient estimate. (*: p<0.05). 2. The X-axis displays different time lags: 1 for two weeks lag, 2 for four weeks lag, and so on. 3. Abbreviations: see Table 1.

Figure 2 displays estimates of regression coefficients of independent variables (Xs) in the logistical regression model for the “occurrence” of indigenous dengue cases. We found that the variables of the number of imported cases (imported, p = 0.0023∼0.0315) and daily maximum/mean/minimum temperatures (tmax/tmean/tmin, p = 0.0002∼0.0495) were positively correlated. On the contrary, relative humidity (rh) was negatively correlated with indigenous case occurrences (p<0.0001∼p = 0.0433). These findings indicate that an increase in imported cases, in conjunction with warmer and drier weather, is favorable for the occurrence of indigenous dengue. Among other meteorological variables, one sunshine related variable and wind speed did not exhibit consistently significant relationships with indigenous dengue (data not shown). However, Figure 3 reveals that, the influence of both imported cases and weather conditions on the “increase” of indigenous dengue was less significant. In addition, when binary outcomes were replaced with indigenous case counts (Figure 4), the quantitative relationships between imported and indigenous dengue cases became insignificant.

### Impact of imported dengue on indigenous dengue at three epidemic phases

**Figure 5:**
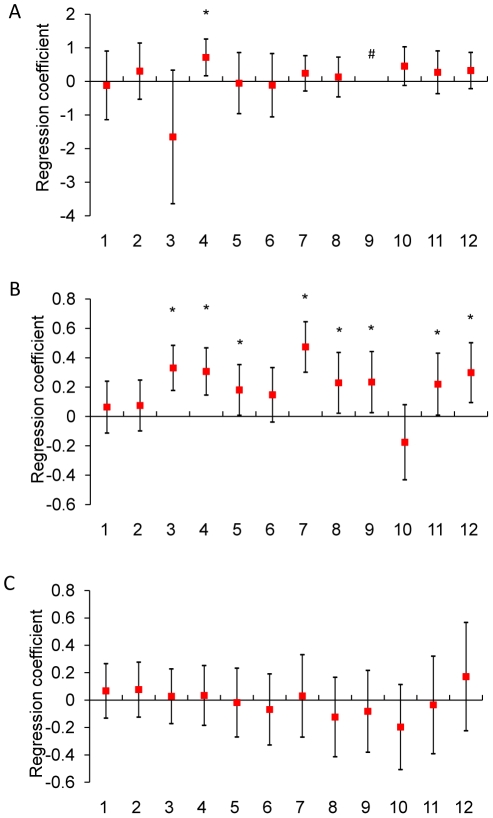
Correlation between bi-weekly number of indigenous dengue cases and the number of imported cases over time lags from 1 to 12 bi-weeks. **A. Period of “low intensity transmission”**: Those bi-week intervals were from March to May. #: 95% Confidence interval = [−419930, 419876.8]. **B. Period of “early phase of outbreaks”**: Those bi-week intervals presenting<10 indigenous dengue cases for months excluding March to May. **C. Period of “late phase of outbreaks”**: Those bi-week intervals presenting> = 10 indigenous dengue cases. Note: 1. Each vertical line segment corresponds to a 95% confidence interval of the regression coefficient and the red solid squares indicate the value of each coefficient estimate. (*: p<0.05). 2. The X-axis displays different time lags: 1 for two weeks lag, 2 for four weeks lag, and so on.

In Figure 5, we observed variation in the impact of imported dengue at different epidemic phases (please see definitions in Methods). Using Poisson models, we found that the imported dengue cases were significantly correlated with indigenous dengue with lag 4 (i.e. 8 weeks) only in **periods of “low intensity transmission”** (Figure 5A). However, this relationship was more statistically significant in the **“early phase of outbreaks”** (Figure 5B). Imported dengue had their greatest impact on epidemics during this phase. When local epidemics entered a p**eriod of “late phase of outbreaks”**, these correlations disappeared (Figure 5C), suggesting that imported cases were unlikely to have influence on indigenous cases during this period. These findings may indicate that imported dengue cases initiate local dengue cases almost exclusively during the onset of an epidemic.

## Discussion

This study examined all laboratory-confirmed dengue cases detected through a combination of active, semi-active, and passive surveillance, and found that imported dengue are able to serve as an initial facilitator, or spark, for domestic epidemics. Nevertheless, imported dengue cases do not have a noteworthy effect from March to May, during the low transmission period of dengue in Taiwan. When these sparks meet suitable weather conditions, the tinder, local dengue epidemics result. Eventually, this relationship disappears once biweekly indigenous case numbers rise over ten, indicating that a local epidemic has occurred. Our findings thus provide evidence that a significant quantitative relationship between Taiwan's imported and indigenous dengue case numbers exists solely at the onset of an epidemic and in the context of appropriate meteorological conditions. Because the numbers of imported dengue cases that initiate indigenous cases have been increasing in non-endemic areas such as Taiwan [4], [5], [6], [8] (further supported by the high nucleotide identities of dengue viruses isolated from travelers with travel history to endemic countries [5], [6], [13], [16], [19]), this study ventures to provide epidemiological evidence of the combined impact of both imported dengue and weather conditions on local outbreaks.

Climate factors have provided helpful clues for monitoring dengue's transmission in affected areas [20], [21], [22], [23]. Higher temperature has the effect of shortening the time intervals of extrinsic incubation in the mosquito life cycle [23], [24] and is positively correlated with more occurrences of indigenous dengue in our study. This is consistent with previous findings that demonstrate the suitability of warm or hot weather for the survivorship of adult mosquitoes and, thus, dengue transmission [22], [25]. In addition, although increased rainfall has been shown to increase the number and quality of mosquito breeding sites (as well as the density of resting sites) [21], lower rainfall and relative humidity (RH) were significantly related to indigenous dengue in this study. The correlation between lower RH and indigenous dengue with time lags was also observed in previous studies in Thailand [26], [27]. We explain this phenomenon as follows. Drier conditions may facilitate dengue transmission through the increase of water storage behavior, which result in an increase of breeding sites for *Aedes* mosquitoes, particularly in areas without reliable water supplies [28], [29], [30]. Although piped water supply is available in 90% of Taiwan (http://www.water.gov.tw/eng/08statistics/sta_a_main.asp?bull_id=4341), water storage for gardening or agricultural use is popular during water restriction period in the dry season, October to April, in southern Taiwan. In addition, a previous field survey identified water buckets as the most common breeding sites of *Ae. aegypti* in southern Taiwan [31]. Entomologically, lower RH (50% vs. 90%) aids higher flight speed of female adult *Ae. aegypti* at temperatures higher than 21 degrees of Celsius [32] thus facilitating dengue transmission. This may explain why both RH and rainfall showed a negative correlation with the number of indigenous dengue (Figure 4) and, that while higher temperatures occurred during July to September in the summer of Taiwan, the number of indigenous dengue cases usually peak in October–November. On the other hand, although the correlation between drier conditions and increased transmission is unlikely to be caused by higher temperatures, we acknowledge that the effects of meteorological factors have a complex relationship. Unlike the consistent negative correlation across lags 3–8 (rain) and lags 4–10 (rainhr) in Figure 4, the positive correlation of “rain” and “rainhr” in Figure 3 occurred only in lag 9, and was therefore most likely a random statistical anomaly rather than a conclusive finding. We believe that weather-based mechanisms that support the proliferation of indigenous dengue therefore need further region-specific investigation and more international collaboration.

To the best of our knowledge, this is the first study to simultaneously identify the relationship between indigenous and imported dengue cases in the context of meteorological factors. Our findings provide a highly accurate epidemiological portrait of dengue in Taiwan because of the following components of the research: First, a better surveillance system was instituted to actively rather than passively detect dengue cases. This system was also laboratory-based to minimize confounding infection and manifestations [5], [13], [33], [34]. Second, we avoided a potential bias as a result of delays in dengue notification by analyzing all confirmed dengue cases in accordance to their onset dates of illness rather than their reporting dates.

We consider that vector control efforts on dengue cases do not affect outbreak initiation, but rather the size and magnitude of an outbreak. A dengue notification delay of over one month allows for two transmission cycles, and increases the potential for a large outbreak [35]. Vector control operations in Taiwan are unlikely to influence imported cases to initiate local dengue epidemics because they are implemented after case notification [10]. By the time indigenous dengue cases increase, the relationship with imported cases disappear (Figure 5C). Hence, the focus of this study was to verify the correlation between imported dengue and the onset of local dengue epidemics under appropriate weather conditions.

In order to construct the best possible regression models to reflect meteorological conditions, we built alternative statistical models to demonstrate the role of imported cases in the onset of dengue epidemics. Previous modeling studies using ARIMA (Autoregressive Integrated Moving Average) found that the number of imported dengue cases was not associated with the incidence of local dengue [9], [36]. ARIMA examined a linear relationship between case numbers of imported dengue cases and incidence of indigenous dengue cases over several time lags. However, the quantitative relationship between imported and indigenous dengue was likely limited to the onset (i.e. early phase) of outbreaks, and was therefore not subject to linear modeling. We believe these conclusions by logistic and Poisson regression models are not only demonstrable in countries with distinct seasonality, but also applicable in non-endemic areas of dengue. However, meteorological conditions may need to be modified for countries in higher altitudes.

Under suitable weather conditions, dengue viruses introduced via travelers are likely to result in further domestic spread and subsequent occurrence of epidemics. In addition, the introduction of more virulent genotypes of dengue viruses has been documented as a potential factor for driving new epidemics [37], [38], [39]. For example, Thai strains belonging to the 1980–1994 clade within the genotype I of dengue virus serotype 1 (DENV-1) were replaced by a 1990–2002 clade [6]. Additionally, an old clade in genotype I of DENV-3 during 1976–1978 was also replaced by a new 1991–2002 clade in genotype II [5], [6]. Furthermore, cosmopolitan genotypes of DENV-2, the causing agent of Taiwan's largest-scale epidemic of dengue/DHF in last thirty years, had been gradually and effectively replacing Asian genotype 2 in the Philippines since 1998 and entered Taiwan in 2001 [40]. This cosmopolitan genotype of DENV-2 is different from the Asian 1 and Asian 2 genotypes of Taiwan's DENV-2 isolates from 1981 to 1998 and the American/Asian genotype of Taiwan's isolates in 2005, when the majority of dengue cases were dengue fever [41]. In other words, the more virulent genotypes/strains of the same serotype that have emerged during later years have resulted in more severe and/or larger-scale epidemics of dengue/DHF in many Asian countries [37], [39].

Based on phylogenetic analyses of dengue viruses isolated from imported cases [6] at the micro-level, we find that local dengue epidemics in Taiwan typically originate in South East Asia. It is therefore imperative to establish a stable surveillance system to detect the spread of different genotypes of DENV. Currently, Taiwan's comprehensive dengue surveillance system is evolving and, hopefully, it may continuously monitor the possible evolution of DENV in SE Asian countries through international collaboration. We believe that global warming may have further impact on the incidence of imported dengue cases and future dengue/DHF epidemics [42]. Advanced research integrating virus displacement and meteorology will be necessary to provide a fuller understanding of both the macro and micro changes contributing to the increasing severity of dengue/DHF epidemics.

This study had notable limitations. First of all, meteorology alone does not initiate an epidemic. Herd immunity also plays a decisive role in the spread of disease. Once a new or more virulent genotype/strain of dengue virus is introduced, public health officials should alert the public and implement prevention efforts regardless of meteorological conditions. Second, local entomological data from Taiwan's entomology surveillance was not included. Non-government scholars do not have access to such data prior to 2002. Furthermore, the data was divided by village or “Li” – the basic administration unit in Taiwan, and was not systematically collected with a standardized process. Therefore, we did not use entomological data because of its lack of consistency and inability to adequately represent the locations covered in our study. Lastly, although socioeconomic status may influence vector habitat [43], it was assumed to be relatively stable during the studied ten years.

As an increase in viremic international travelers has caused global DHF case numbers to surge in the past several decades [44], efficient measures have to be instituted to prevent imported dengue cases from igniting local dengue/DHF epidemics. Additionally, it has been previously found that DHF cases with higher viral load [45] appeared when the number of dengue fever cases increased rapidly, particularly in areas with higher dengue clusters [46]. All these findings suggest that the entrance of imported cases, in conjunction with suitable meteorological conditions, may have the potential to precipitate severe epidemics involving more DHF cases. Careful tracking and clinical management of imported dengue cases, along with relevant meteorological information, are able to provide earlier warning signals for emerging indigenous dengue epidemics than current surveillance systems [11], [20]. These early alerts allow for the proper implementation of targeted public health interventions and valuable buffer time for preventing subsequent large-scale epidemics of dengue/DHF locally and in affected countries. Advanced investigation and integration of immunological, virological, meteorological, and entomological findings with prevention/control strategies will support a more comprehensive understanding of the mechanisms that initiate dengue epidemics, and will help guide realistic public health interventions in the era of rapid globalization and climate change [47].

## Supporting Information

Alternative Language Abstract S1 Translation of the Abstract into Chinese by C-S Shang.
(0.04 MB DOC)

Text S1 Content of regression models.
(0.03 MB DOC)
