# Supplementary material for: The Role of Imported Cases and Favorable Meteorological Conditions in the Onset of Dengue Epidemics
Source: PLoS Negl Trop Dis. 2010 Aug 3;4(8):e775. doi: 10.1371/journal.pntd.0000775 (PMC2914757; doi:10.1371/journal.pntd.0000775)
Supplement: Alternative Language Abstract S1 — Translation of the Abstract into Chinese by C-S Shang. (0.04 MB DOC) [file pntd.0000775.s001.doc]

境外移入病例和適合的氣象條件在登革流行初期的角色

尚君璽^1^, 方啟泰^1, 2^*, 柳中明^3, 4^*, 溫在弘^5^, 蔡坤憲^1^, 金傳春^1^*

^1^國立臺灣大學公共衛生學院流行病學研究所（中華民國臺灣，以下同）. ^2^國立臺灣大學附設醫院內科. ^3^國立臺灣大學全球變遷中心. ^4^國立臺灣大學理學院大氣科學系. ^5^國立臺灣大學理學院地理系.

*作者對本文有相同的貢獻

**機構地址：**（100）臺北市徐州路17號
國立臺灣大學公共衛生學院流行病學研究所

**本文回覆作者電子郵件信箱：** [chwanchuen@gmail.com](mailto:chwanchuen@gmail.com)（金傳春）與 [fangct@ntu.edu.tw](mailto:fangct@ntu.edu.tw) （方啟泰）

**中文摘要**

**背景**

受登革病毒感染的旅客經常成為散播病毒至其他地區的重要途徑，甚而引發他國的流行；然而這些感染的旅客入境後和當地氣候、病媒以及本地疫情之間的互動關係並不清楚。由於境外移入病例與本地登革疫情關係的議題長期受到忽略，本研究即在探究臺灣地區的境外移入登革病例和氣候因子，對於本地疫情發生之影響。

**方法與主要發現**

我們使用羅吉斯（logistic）和普瓦松（Poisson）迴歸模式分析1998至2007年間臺灣南部地區經實驗室診斷證實的登革確定病例，以區辨在氣候因子的作用下，境外移入和本地登革病例的時序相關性。結果發現本地登革疫情的發生與境外移入病例數（2至14週前）、高溫（6至14週前）及低濕度（6至20週前）之間，存在延遲的相關性。此外，境外移入病例數和本地登革病例數僅在流行被引發的初期階段，才有明顯數量上的相關性；一旦流行持續發生，此種關係即不復見。

**結論**

這些發現顯示，惟有氣象條件適宜時，境外移入登革病例才有可能引發本地的疫情。據此，經由境外移入病例的快速實驗診斷、早期發現以及管理，可以遏止其後大規模登革/登革出血熱流行的發生。因此整合氣象資訊的早期警示監測系統，將是登革疫情尚未成為地方性流行的地區用以成功防治疫情的無價利器。
