# Supplementary material for: The Role of Imported Cases and Favorable Meteorological Conditions in the Onset of Dengue Epidemics
Source: PLoS Negl Trop Dis. 2010 Aug 3;4(8):e775. doi: 10.1371/journal.pntd.0000775 (PMC2914757; doi:10.1371/journal.pntd.0000775)
Supplement: Text S1 — Content of regression models. (0.03 MB DOC) [file pntd.0000775.s002.doc]

**Text S1: The models used in this study are listed below.**

1. Modeling the temporal correlation between “Occurrence” of indigenous dengue and variables: Occurrence (y/n) ~ sin24 + cos24 + two dummy variables for areas+ population density + tested lagged variable, link = logistic, family = binomial
2. Modeling the temporal correlation between “Increase” of indigenous dengue and variables: Increase (y/n) ~ sin24 + cos24 + two dummy variables for areas+ population density + tested lagged variable, link = logistic, family = binomial
3. Modeling the temporal correlation between the number of indigenous dengue and variables: Case (count) ~ sin24 + cos24 + two dummy variables for areas+ population density + tested lagged variable, link = log-linear, family = negative binomial
4. Modeling the temporal correlation between the number of indigenous and imported dengue in Period of “low intensity transmission” (Those bi-week intervals were from March to May):
   Case (count) in Period of “low intensity transmission”~ sin24 + cos24 + two dummy variables for areas+ population density + tested lagged variable, link = log-linear, family = Poisson
5. Modeling the temporal correlation between the number of indigenous and imported dengue in Period of “early phase of known outbreaks” (Those bi-week intervals presenting <10 indigenous dengue cases for months excluded March to May):
   Case (count) in Period of “early phase of known outbreaks”~ sin24 + cos24 + two dummy variables for areas+ population density + tested lagged variable, link = log-linear, family = Poisson
6. Modeling the temporal correlation between the number of indigenous and imported dengue in Period of “late phase of known outbreaks” (Those bi-week intervals presenting≧10 indigenous dengue cases):
   Case (count) in Period of “late phase of known outbreaks”~ sin24 + cos24 + two dummy variables for areas+ population density + tested lagged variable, link = log-linear, family = negative binomial
